# Aggressiveness, Mating Behaviour and Lifespan of Group Housed Rabbit Does

**DOI:** 10.3390/ani9100708

**Published:** 2019-09-20

**Authors:** Zsolt Gerencsér, Zsolt Matics, Rubina T. Szabó, Károly Kustos, Annamária Mikó, István Nagy, Meinrad Odermatt, Tamás Atkári, Zsolt Szendrő

**Affiliations:** 1 Faculty of Agricultural and Environmental Sciences, Kaposvár University, 7400 Kaposvár, Hungary; matics.zsolt@ke.hu (Z.M.); annamarimiko@gmail.com (A.M.); nagy.istvan@ke.hu (I.N.); szendro.zsolt@ke.hu (Z.S.); 2 Faculty of Agricultural and Environmental Sciences, Szent István University, 2100 Gödöllő, Hungary; szabo.rubina@mkk.szie.hu (R.T.S.); kustos.karoly@mkk.szie.hu (K.K.); 3 Olivia Ltd, Mizse 94, 6050 Lajosmizse, Hungary; modermatt@delimpex.ch (M.O.); tamas.atkari@olivia.hu (T.A.)

**Keywords:** group housing, rabbit does, aggressive behaviour, mating behaviour, rank order, lifespan

## Abstract

**Simple Summary:**

Aggressiveness is a well-known trait in different animal species, including the European wild and domesticated rabbit. It is one of the main problems in group housing of rabbit does. The aim of the experiment was to investigate the frequency of aggressiveness and mating behaviour as well as the lifespan of does depending on their rank order. Dominance hierarchy developed during aggressive behaviour, i.e., fighting. In most cases the primary position in the rank order was clear. Mating activity was very high after assembling the groups. In addition to male–female mating, some female-female and female-male mounting was also observed, which could lead to pseudo-pregnancy. A second small peak of sexual activity was observed at the end of the hypothetical pseudo-pregnancy. Based on the results it can be concluded that aggressive behaviour is frequent in group housing systems which is contrary to animal welfare. Natural mating is not effective in group-housing systems.

**Abstract:**

Aggressiveness is one of the main problems in group housing of rabbit does. The aim of the experiment was to investigate the frequency of aggressiveness and mating behaviour as well as the lifespan of does depending on group composition. The female and male rabbits were housed in one of the 7.7 m^2^ pens (four females and one male per pen). Based on the ages of female rabbits two homogenous groups (HOM) were formed containing four 17-week-old females and two heterogeneous groups (HET) containing three 17-week-old and one 1-year-old female. Twenty-four-hour video recordings were taken during the first month after assembling the groups, and the aggressive actions (fights) and matings were counted. The lifespan was examined over a 200-day experimental period. On the day after assembling the groups the number of fights among does was high in HET group. The same aggressive behaviour only started a week later in HOM group, and some fights between females and the male were also observed. The daily peaks of aggressiveness were in the morning (after the light on) and in the evening (before and after the lights off). The primary position of females in the hierarchy was clear but sometimes no differences were detected among the subordinate females. The mortality of does was connected with their rank order. The number of matings was very high on the day of assembling the groups and a second small peak was observed at the end of the hypothetical pseudo-pregnancy. In addition to mating between male and females, female–female and female–male mounting was also observed. Despite of the small sample size it seems that aggressive behaviour is frequent in group housing systems, which is contrary to animal welfare. Natural mating is not effective in group-housing system.

## 1. Introduction

In some European countries (Netherland, Belgium, etc.) the group-housing system of rabbit does is recommended. The main purpose of the group-housing system is to provide conditions for rabbit does as close to nature as possible. Results of group housing of rabbit does were reviewed by some authors [1,2,3]. In these papers the negative/positive consequences of group housing are well presented. According to these articles one of the main problems in group housing systems is aggressiveness, i.e., fighting and injuries. On Swiss farms 33% of does had at least one lesion and the occurrence of more severe injuries was 9% [4]. In another study the percentage of injured does was 17% and 21% [5], respectively. In the experiments when the does were housed together continuously, the aggressive behaviour declined after stabilization of a social rank order [6]. After stabilizing the social conditions, subordinate females frequently showed submission to dominant counterparts [6]. Aggressiveness caused the stress hormone level to be higher in does housed in groups compared to those housed individually [7].

Aggression is a common behaviour in animal species. Animals often use aggressive behaviour to defend a resource, to compete for mates, to defend against predators or during foraging. Territorial animals defend their territories against intruders [8]. Aggressiveness is also common in both wild and domesticated rabbits [9]. 

The ancestor of the domestic rabbit, the European wild rabbit (*Oryctolagus cuniculus*) lives in territorial breeding groups, which consist of about 2 to 3 bucks, 2 to 9 does, and their kits [10]. The aggressiveness of wild rabbit females starts at the beginning of breeding season, and the frequency of fighting declines after group stability is established [11,12]. Female and male wild rabbits form independent linear hierarchies, and often an older animal is in the dominant position of a group [6,12,13]. Fighting between females is most pronounced at the start of the reproductive season [5]. After stabilizing the order of hierarchy, aggressive chasing and fighting is rare [13]. Aggression is also a common problem in the case of sows which are housed in groups. It occurs mostly because of competition for access to a limited resource, or to establish a social hierarchy [14]. The aggression related to competition for a place in the social hierarchy can be highly intensive. The whole process can take from 2 to 10 days after assembling the groups, and in general, older sows are more dominant [14].

Based on the results of the experiment the kindling rate of rabbits is low in group-housing systems [1,5,15], while the rate of pseudo-pregnant does is high (23 %) due to the sexual attempts that were observed among the does [5]. 

The suckling mortality was twice as high in group-housed does as in individually housed ones [7,16]. One of the reasons was that in some cases two or three does kindled into the same nest box [7,16]. The nest boxes were used as a resting place or does escaped there from the aggressive animals [17]. Survival of rabbit does was lower in groups than in individual housing [7].

In nature the reproductive success and lifespan of females depends on their social rank [12]. High position in rank order is often associated with fitness benefits for individuals [12]. During the breeding season the dominant doe produced more litters than the subordinate ones, and the survival rate of kits was highest for those of the dominant ranking doe and lowest for those of the lowest ranking doe [13]. According to an observation the number of litters, number of kits and number of adult offspring per year significantly decreased between social ranks 1 and 5 [12]. Similarly to rabbits, high-ranking sows had a higher farrowing rate and produced more piglets than the low-ranking animals in the same group [18]. However, the results in literature describing the effect of rank position on the reproductive performance are contradictory. 

Mortality of rabbit does also showed a connection with their rank position, a higher survival rate of rabbits was observed in higher position [12]. Mortality of kits increased in litters of low-ranking females [19]. Infanticide was also observed in group housing systems [19]. Its proportion was higher in groups where same-age females were grouped together than in groups where the females’ rank hierarchy had a more heterogeneous and linear age structure. Even though more observations have been conducted into the social behaviour of European wild rabbit, little is known about group-housed rabbit does. 

Researchers mainly focused on the production and welfare aspects of group housing, while there are no observations regarding the hierarchy, mating behaviour and their effect within the group. According to the observation of Rödel et al. [20] the frequency of aggressive behaviour would be reduced if there was an older female in the group after her position as the dominant animal was established as this would only take a short period of time. Based on this the aim of this experiment was to evaluate the aggressive and mating behaviour, as well as the lifespan of group housed does under different group compositions (with or without an older female in the group).

## 2. Materials and Methods

### 2.1. Animals and Experimental Design

The experiment was conducted at the Kaposvar University using maternal line (Pannon Ka) rabbit does of the Pannon Rabbit Breeding Program [21]. Rabbits were housed in pens which were constructed according to the recommendation of an animal protection group (Vier Pfoten). In this system four female rabbits and one male were continuously kept together in a pen, with a basic area of 7.7 m^2^. Within the pen, half of the basic area was covered with straw, whereas the other part was made of plastic-mesh. Every pen was equipped with a large sized feeder, five nipple drinkers, hay rack, four wooden nest boxes and a plank tube for hiding. The female and male rabbits were selected for the experiment based on their health and condition. From this viewpoint the groups were balanced. The pens were located in two identical experimental rooms. The temperature in the rooms was 15–17 °C. Basically natural light was used, which was supplemented with artificial lighting to 16 h period of light per day. Rabbits received a commercial pellet ad libitum and they had free access to hay from the hay rack. Water was available ad libitum from nipple drinkers.

Based on the ages of female rabbits two homogenous groups (HOM1 and 2) were formed containing four 17-week-old females (*n* = 8) and two heterogeneous groups (HET1 and 2) containing three 17-week-old (*n* = 6) and one 1-year-old female (*n* = 2) per group.

### 2.2. Video Recording

Infra-red cameras were fixed above the pens. Continuous 24-hour video recordings were made during 30 days after assembling the groups. The fur of rabbits was marked with different notations (like spots, lines, crosses) which were made with black hair dye. This method was necessary to follow the behaviour of rabbit does individually. All aggressive behaviour (the attacker and attacked animals), and the mating behaviour of the does and bucks (mating between buck and doe or between two does) were recorded. From the data of initiated and suffered aggression events a 4 × 4 “winner–loser” matrix was generated in each group. Based on Hoy et al. [18] from this data rank index (RI) was calculated using the following formula:

RI = (IA × P_IA_ − SA × P_SA_)/((IA + SA) × (n − 1))

where,

IA = number of initiated aggression events;
P_IA_ = number of attacked partners;
SA = number of suffered aggression events;
P_SA_ = number of partners which initiated attack;
*n* = group size.

Based on the value of rank index a social rank order was determined in each group.

Daily and hourly events were also evaluated. The connection between the social rank order and lifespan during a 200-day period was also evaluated.

### 2.3. Statistical Analysis

Aggression, total and successful matings were analysed using GLM analysis. In the first analysis group composition (HOM1, HOM2, HET1, and HET2) and days (1–30) were treated as fixed factors. In the second analysis hours (1–24) were considered as fixed factor instead of days, while group composition remained the same as before. Group and day and group and hour interactions were also analysed by means of one factor GLM analysis where the group levels were reduced to two (the HOM1 and HOM2 and the HET1 and HET2 groups were merged). Different set of means were compared using orthogonal contrasts. All statistical analysis was performed using SAS 9.4 software.

## 3. Results

### 3.1. Aggressive Behaviour, Dominance Rank Order

During the month after assembling the groups the LS means of aggressive attempts were 5.0, 2.3, 3.8, and 4.3 in groups HOM1, HOM2, HET1 and HET2, respectively which were not significantly different (*P* = 0.06). Our hypothesis, which was based on the observation of Rödel et al. [20] was that the frequency of aggressive behaviour would be reduced if there was an older female in the group because after a short period she would become the dominant animal. However, this theory was not confirmed.

**Figure 1:**
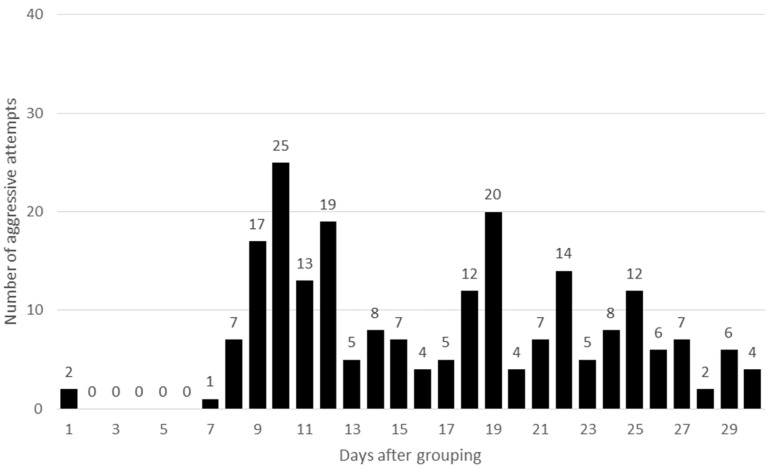
Cumulative number of aggressive behaviours in homogenous (HOM) groups.

**Figure 2:**
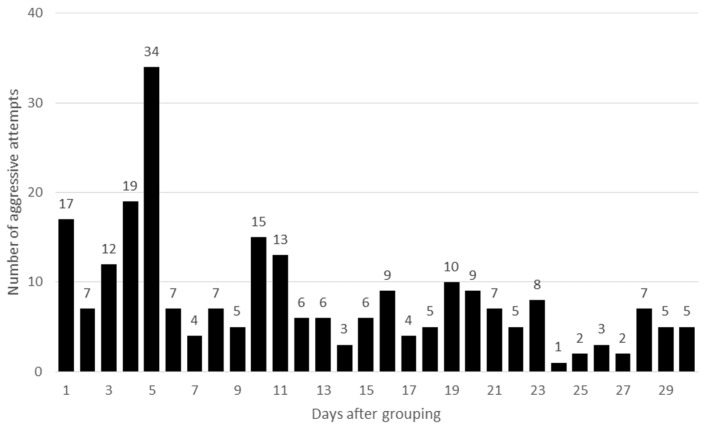
Cumulative number of aggressive behaviours in heterogeneous (HET) groups.

In both HOM groups only a few aggressive interactions were observed in the first week, while in HET groups the fighting started right after the groups were formed (Figure 1 and Figure 2). Differences were significant between the HOM and HET groups both for the first day (*P* = 0.04) and for the first week (*P* < 0.001), respectively. It seems, that if the age of female rabbits was the same (HOM group), the aggressive interactions for a higher rank position started later. The frequency of aggressiveness was higher in the second week in HOM group compared to the HET group (*P* = 0.05). There were no further significant differences for the rest of the analysed period (*P* > 0.05). In European wild rabbit at the beginning of the reproductive season fights were very intense among females but later their intensity decreased [22]. In semi-group housing systems aggression and fighting were very frequent after each re-grouping but decreased after a few days [6,15,22]. In the present experiment a definite decline was only observed in one group (HET2; (data are not shown). In the other groups the decrease was not so pronounced. Zomeño et al. [23] observed that the aggressive interactions were high on the first day of group formation, mainly during the first few hours. However, Rommers et al. [17] also observed small differences in the frequency of doe injuries between days 4 and 17 after being housed in groups. It seems that in the present experiment, with the exception of one group, aggression could be continuously observed with variable amplitudes during the whole examined one-month period.

**Figure 3:**
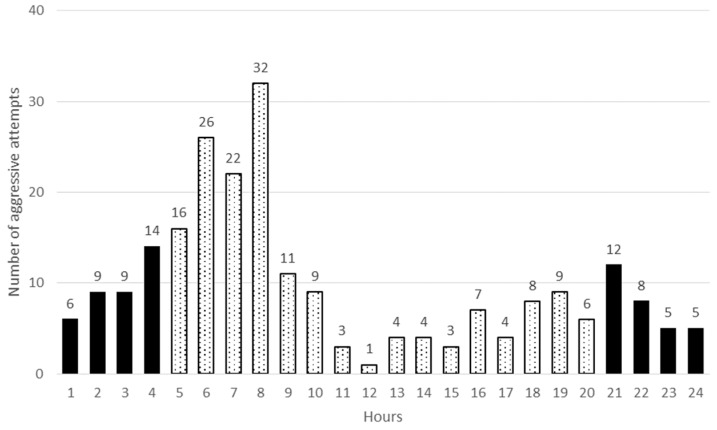
Number of aggressive interactions in relation to time of day in HOM groups (light and dark columns show the light and dark periods of day).

**Figure 4:**
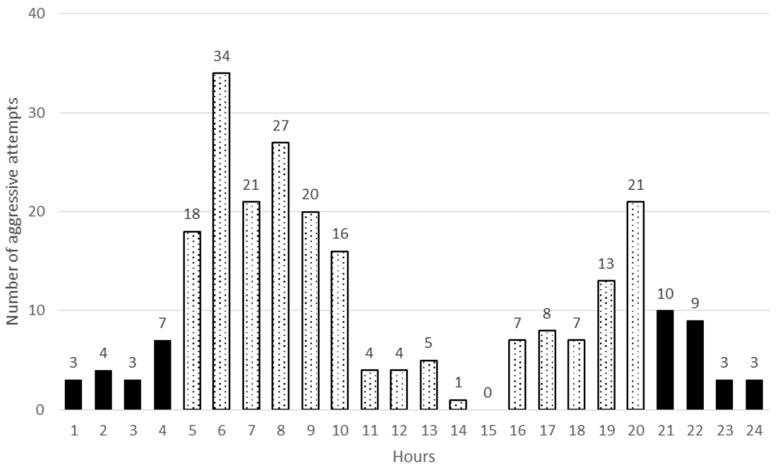
Number of aggressive interactions in relation to time of day in HET groups (light and dark columns show the light and dark periods of day).

Aggressive interaction among does was observed both in dark and light periods of the day. There were two peaks of aggression, one in the morning (after the light on, between 6 and 8 h) and the other in the evening (before and after the lights off, between 19 and 21 h) in both groups. No significant differences were detected between the HOM and HET groups neither in the morning nor in the evening (*P* > 0.05). However, much higher frequencies were observed in the morning (Figure 3 and Figure 4). The daily fluctuation in aggressive behaviour could be in close connection with the nocturnal activity of rabbit. Several years ago, Southern [24] observed that wild rabbits were most active around dawn and dusk, which was later observed by several other researchers (e.g., [25,26]). Myers and Poole [27] also recorded aggressive behaviour in the morning. 

The dominance rank order was established based on the value of the rank index (Table 1, Table 2, Table 3 and Table 4). It can be seen that the Doe1 and Doe3 were the first ranking animals in HOM1 and HOM2 groups, respectively (Table 1 and Table 2). The rank index of the animals with position 1 and 2 in HOM1 group, and with position 2, 3, and 4 in HOM2 group, were similar. In a few cases the male rabbits were the attackers (2 and 3 times in group HOM1 and HOM2, respectively, while they themselves were attacked 8 and 5 times in groups HOM1 and HOM2, respectively; (data are not shown in the tables).

In the HET1 group the rank order is very clear, the older doe (Doe1) was the dominant animal with a high value of rank index, followed by the Doe2, Doe3, and Doe4 (Table 3). It seems that the dominant rabbit was extremely aggressive. There were also some fights between the buck and Doe1, of these, 4 times Doe1 and 3 times the male rabbit was the attacker (data are not shown in the Table 3).

A clear dominance rank order was established in group HET2 (Table 4), however the older female (Doe1) was in second position. Sometimes the male attacked Doe1 and Doe3 but Doe3 and Doe4 also attacked the male rabbit (data are not shown in the Table 4).

According to Mykytowycz [13] and von Holst et al. [12,22] the social hierarchies are based on the fights among wild rabbits, and linear ranking orders are established separately among male and female wild rabbits. In the present study a few fights were observed between males and females in similar frequencies: In 1%–5% of cases the buck attacked one of the does and in 3%–8% of cases one of the does attacked the male. However, in case of limited space, Cowan [28] and Mykytowycz and Hesterman [9] also observed some intersexual aggression.

Generally, in their first breeding season wild female rabbits occupy a lower rank position and older females are the dominant [12,20,22]. However, according to von Holst et al. [12] 8% of females gained the dominant position in the first season. In the present study the older does in HET groups were in the first and second position, which is consistent with the authors’ observations.

In the experiments where groups of does were assembled [4,5,15,17,29] their injuries were counted, however the fighting among does and the dominance rank order was not examined.

According to von Holst et al. [12] there is a close correlation between the rank position of wild female rabbits and their lifespan because all females in social rank position 4 and 6 died before their second reproductive year. Although there were few rabbits and the length of the present study was short (200 days), the mortality results only partially confirmed this finding. It is very interesting that 75% of the Rank1 and Rank4 rabbits died and mortality among Rank2 and Rank3 females was only 25%. Based on these results it seems that maintaining the first position could cause a very stressful situation in this system. Except one female (in Rank1 position) who died during a fight (perhaps because of a spine injury), all rabbits were lean and in bad condition before they died, which could be attributed to chronic stress. 

### 3.2. Mating

The LS means of the total mating attempts of buck per day were 6.3 and 14.4 in HOM1 and HOM2 groups and 1.8 and 3.6 in HET1 and HET2 groups, respectively. The LS means of the successful matings per day were 1.97, 2.10, 0.37, and 0.80 in HOM1, HOM2, HET1, and HET2 groups, respectively, in the same period. Both the total and successful mating attempts were higher in HOM groups than in HET groups (*P* < 0.001 and *P* = 0.04). It can be supposed that the higher successful mating attempts were higher in the HOM groups because there were less aggressive interactions, hence less disruptions or less stressful episodes in these groups compared to HET.

**Figure 5:**
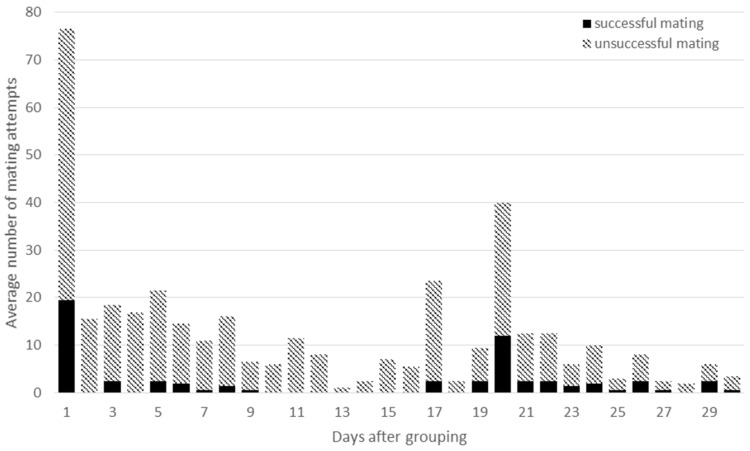
The average number of successful matings (dark column parts) and unsuccessful mating attempts (light column parts) in HOM groups during the first month after assembling the groups.

**Figure 6:**
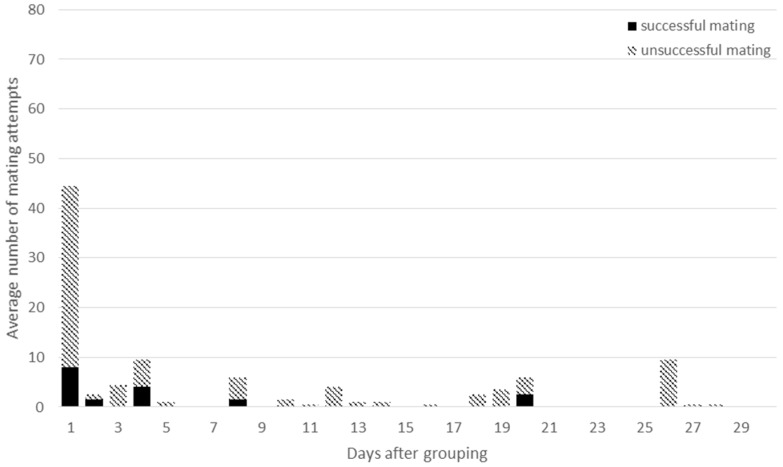
The average number of successful matings (dark column parts) and unsuccessful mating attempts (light column parts) in HET groups during the first month after assembling the groups.

On the first day after assembling the groups the average number of successful and total mating attempts per group were 20 and 57 in HOM groups, and 8 and 36 in HET groups, respectively. The differences between the HOM and HET groups were significant both for the successful and for the total matings (*P* < 0.001 and *P* = 0.02; Figure 5 and Figure 6). In the next two weeks the total number of mating attempts decreased with fewer events in the HET groups. Significant difference was only found between the HOM and HET groups for the total number of matings during the first week (*P* < 0.001). However, higher values were observed again between days 17 and 20, when the period of a pseudo-pregnancy ends. During this period significant differences were observed between the HOM and HET groups for the total and successful matings, respectively (*P* = 0.002, *P* = 0.02). At the same time, several mating attempts were observed between two females and between female and male, mainly after assembling the groups. It seems that male rabbits were very active on the first day, but they were not able to mate with all females in heat. This is why mating behaviour was observed between two females and between female and male, which could cause pseudo-pregnancy. It generally lasts 16 to 18 days [30], after which period the progesterone concentration decreases to basic level [31]. High numbers of mounts were reported by Rubin and Azrin [32] and Jimenez et al. [33]. Beyer and Rivaud [34] observed a second peak of mounting in the last trimester of the pregnancy of rabbit does. At the end of pseudo-pregnancy Caillol et al. [35] observed lordosis and accepted mating and most females were receptive. Rommers et al. [5] found 23% pseudo-pregnant does in group housing system. Pseudo-pregnancy can result from sterile mating, however in the present experiment false mating attempts were observed between females and some females also wanted to mount the buck. Female–female mounting was also observed by Albonetti et al. [36] in different ratios in the examined groups. Comparing the HOM1 and HOM2 groups, similar tendencies can be seen (data are not shown). 

**Figure 7:**
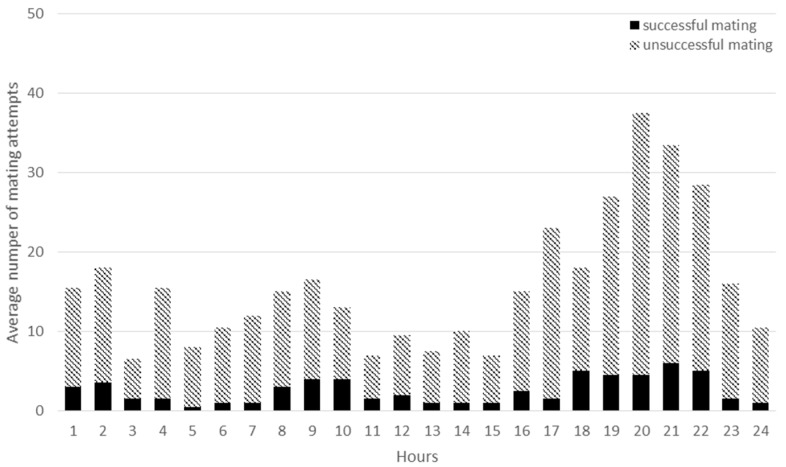
The daily distribution of successful matings (dark column parts) and unsuccessful mating attempts (light column parts) in HOM groups.

**Figure 8:**
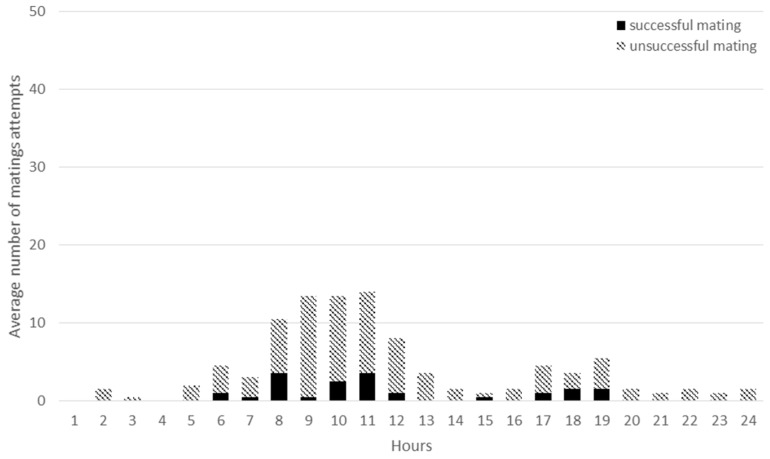
The daily distribution of successful matings (dark column parts) and unsuccessful mating attempts (light column parts) in HET groups.

There was a definite difference in daily mating frequency between HOM and HET groups (Figure 7 and Figure 8). In the HOM group the total and successful matings were higher than in the HET group between 17:00 and 22:00 hours (*P* < 0.001). In the HET group it was most frequent between 8:00 and 12:00 but the differences were not significant compared to the HOM group (*P* = 0.65, *P* = 0.29). European wild rabbits generally leave their burrows around dusk, are active throughout the night, and return to their burrows in the early morning [37]. Villafuerte et al. [38], Diez et al. [25] observed an increased activity during twilight and night, with two peaks at sunrise and sunset. According to Jilge [39] under laboratory conditions rabbits are nocturnally active animals, but this can be modified by external factors. It may be assumed that in the present experiment the difference between the two groups can be traced back to the fact that the groups were formed in the morning and the mating activity was very high after assembling the groups. However, this explanation only seems to be justified in the case of HET groups, and does not explain the difference between HOM and HET groups.

It seems that there was not a real resting time in the middle of the day because only the frequency of mating decreased. This could be in connection with the high mating activity on the first day of group formation.

The data relating to how many times does were mated either successfully or unsuccessfully, and which doe initiated sexual relations with the other does was collected in a matrix. The matings between male and female animals cannot be evaluated, because if a doe became pregnant, she might have refused the male. At the same time a definite order was observed when one female had sexual connection with another female. The average number of sexual contacts were 23, 18, 4, and 2 when the initiating females were in Rank1 to 4, respectively.

The successful post-partum mating was frequent, but in general there was no relationship found neither in this trait nor in the number of kindlings. At the same time there is a connection between the position in the social rank order and the productivity (number of litters, new-born and weaned kits) of wild rabbit does [12].

## 4. Conclusions

There were many similarities between the social behaviour of European wild rabbits and group-housed rabbit does. Dominance hierarchy was developed during the aggressive behaviour (fighting). The number of fighting incidents was not influenced by the composition of the groups (the age of does was similar or different), however some fighting was also observed between females and males. In most cases the first position in the rank order was clear. The mortality of females was related to the rank order. Mating activity was very high after assembling the groups. In addition to male–female mating some female–female and female–male mounting was also observed which could be related to pseudo-pregnancy. A second small peak of sexual activity was observed at the end of the hypothetical pseudo-pregnancy. Despite of the small sample size it seems that the aggressive behaviour is frequent in group housing systems which is contrary to animal welfare and natural mating is not effective in group-housing system.

## Figures and Tables

**Table 1:** Dominance rank order based on the number of fights among the does (HOM1).

Attacked	Attacker				
	Doe1	Doe 2	Doe 3	Doe 4	Total
Doe1	-	3	7	6	16
Doe2	18	-	28	25	71
Doe3	51	2	-	4	57
Doe4	8	0	2	-	10
Total	77	5	37	35	154
Rank Index	0.66	−0.89	−0.29	0.63	
Rank order	1	4	3	2	

**Table 2:** Dominance rank order based on the number of fights among the does (HOM2).

Attacked	Attacker				
	Doe1	Doe 2	Doe 3	Doe 4	Total
Doe1	-	1	18	5	24
Doe2	0	-	5	2	7
Doe3	6	5	-	1	12
Doe4	13	0	5	-	18
Total	19	6	28	8	61
Rank Index	−0.26	−0.05	0.40	−0.15	
Rank order	4	2	1	3	

**Table 3:** Dominance rank order based on the number of fights among the does (HET1).

Attacked	Attacker				
	Doe1	Doe 2	Doe 3	Doe 4	Total
Doe1	-	0	1	3	4
Doe2	3	-	0	2	5
Doe3	30	2	-	0	32
Doe4	59	5	3	-	67
Total	92	7	4	5	108
Rank Index	0.93	0.11	−0.52	−0.88	
Rank order	1	2	3	4	

**Table 4:** Dominance rank order based on the number of fights among the does (HET2).

Attacked	Attacker				
	Doe1	Doe 2	Doe 3	Doe 4	Total
Doe1	-	1	33	0	34
Doe2	17	-	9	12	38
Doe3	12	2	-	2	16
Doe4	13	1	21	-	35
Total	42	4	63	14	123
Rank Index	0.25	−0.81	0.59	−0.52	
Rank order	2	4	1	3	
